# Old and new coronaviruses in the elderly

**DOI:** 10.18632/aging.203065

**Published:** 2021-05-12

**Authors:** Francesco Nicoli, Deepak Paudel, Maria Teresa Solis-Soto

**Affiliations:** 1 Laboratory of Biochemistry, Immunology and Microbiology, Department of Chemical, Pharmaceutical and Agricultural Sciences, University of Ferrara, Ferrara 44121, Italy; 2 Nepal Public Health Association, Kathmandu, Nepal; 3 Instituto de Ciencias de la Salud, Universidad de O’Higgins, Rancagua, Chile

**Keywords:** SARS-CoV-2, COVID-19, HCoV, T cells, immunosenescence, cross-reactive memory

Since the initial spread of the severe acute respiratory syndrome coronavirus 2 (SARS-CoV-2) from Wuhan in late 2019, early data revealed that infection severity increases with age. Higher mortality rates have been reported among older age groups irrespective of the country’s economy, population structure and other contextual factors [1].

Recent studies detected SARS-CoV-2−reactive CD4^+^ T cells in a relatively high proportion (up to 60%) of unexposed individuals [2,3], most probably due to previous exposure to Common Cold Coronaviruses [3]. It has been hypothesized that these subjects with high levels of pre-existing memory cells cross-recognizing SARS-CoV-2 could set up a faster and stronger immune response and thus control coronavirus disease 2019 (COVID-19) [3]. Data showing that individuals with SARS-CoV-2 acute infection display lower T-cell responses to Common Cold Coronaviruses compared to recovered patients or healthy individuals would concur with this hypothesis [4]. However, it should be noted that acute SARS-CoV-2 infection dampens cellular immunity against heterologous pathogens [4], probably reflecting a transient lymphopenia. This would rather suggest that Common Cold Coronavirus-specific T-cell responses are suppressed during acute SARS-CoV-2 infection, and not that low cellular immunity against HCoV contributes to poor COVID-19 outcomes.

Coronaviruses have been identified in animals (including bats and chickens) and humans and can cause different diseases, spanning from cold (the most frequent manifestation) to gastrointestinal and severe respiratory illnesses. Common Cold Coronavirus includes subtypes of α-coronaviruses (HCoV-229E and HCoV-NL63) and β-coronaviruses (HCoV-OC43 and HCoV-HKU1) and were isolated nearly 50 years ago. These are endemic in humans and have been estimated to cause 15 to 30% of upper respiratory tract infections in adults each year. Common Cold Coronaviruses are more prevalent in adults [5], increasing the seroprevalence with age [5]. Thus, older adults have been exposed to different HCoVs and probably have been re-infected several times. Their immune memory to Common Cold Coronaviruses should be, in theory, strong. Nevertheless, older age groups present with the highest mortality rates from COVID-19, arguing against a protective role of cross-reactive memory responses. Indeed, if the presence of Common Cold Coronavirus-specific memory T cell limits disease severity after infection with SARS-CoV-2, we would expect a low mortality rate in individuals exposed several times to the different HCoVs, i.e. the elderly. Also, it has been shown that re-infection with the same Common Cold Coronavirus is common, even 12 months after the primary infection, which indicates a temporary-limited protective immunity [6]. Therefore, it is questionable that memory responses unable to prevent the re-infection with the same virus would protect against virions of the same family but with relatively low homology.

Conversely, it has been shown that low numbers of naïve CD8^+^ T cells are associated with the risk of severe COVID-19 [7]. This cell subset, which is deputed to mount *de novo* responses against unencountered antigens, such as emerging infections and thus SARS-CoV-2, quantitively decreases with advancing age [1]. Similarly, a functional decline characterizes old naïve T cells [1]. We thus initially hypothesized that the age-associated decline of naïve CD8^+^ T cells could significantly contribute to the susceptibility of elderly individuals to severe COVID-19 [1]. According to this hypothesis, older subjects harbor few and dysfunctional naïve CD8^+^ T cells (including those specific to SARS-CoV-2) and will therefore mount poor virus-specific primary cellular responses, partially or totally unable to control the infection. Consistently, immunomonitoring of infected patients has revealed an inverse correlation between age and SARS-CoV-2-specific CD8^+^ T-cell responses [8].

The loss of naïve T cells occurring with aging is, likely, one of the strongest contributors to the increased incidence of tumors in the elderly and to the high mortality rates that emerging infections show in this population. Transformed cells appearing in the body or emerging pathogens never encountered before represent new threats for the host. The human immune system attempts to neutralize them through naïve lymphocytes. However, whether this cell subset is lost or dysfunctional, such as during the immunosenescence process, the individual is at greater risk of succumbing to the new threats. It is therefore likely that a similar age pattern as characterized in the current pandemic will also be observed for future ones. The same immunological mechanisms dictate the onset of immune response to newly administered vaccines, whose efficacy is generally low in the elderly. This implies that the most vulnerable populations are also more difficult to protect. Consequently, it is urgent to invest in further research that aims at preserving the naïve T-cell pool in the aging population.
